# Early platelet dysfunction in patients receiving extracorporeal membrane oxygenation is associated with mortality

**DOI:** 10.1007/s11239-021-02562-9

**Published:** 2021-09-16

**Authors:** Patrick Malcolm Siegel, Julia Chalupsky, Christoph B. Olivier, István Bojti, Jan-Steffen Pooth, Georg Trummer, Christoph Bode, Philipp Diehl

**Affiliations:** 1 Department of Cardiology and Angiology I, University Heart Center Freiburg – Bad Krozingen, Faculty of Medicine, University of Freiburg, Freiburg, Germany; 2 Department of Cardiovascular Surgery, University Heart Center Freiburg – Bad Krozingen, Faculty of Medicine, University of Freiburg, Freiburg, Germany

**Keywords:** Extracorporeal membrane oxygenation, Mortality, Platelets, Hemorrhage, Critical care

## Abstract

Extracorporeal membrane oxygenation (ECMO) is used for patients with cardiopulmonary failure and is associated with severe bleeding and poor outcome. Platelet dysfunction may be a contributing factor. The aim of this prospective observational study was to characterize platelet dysfunction and its relation to outcome in ECMO patients. Blood was sampled from thirty ECMO patients at three timepoints. Expression of CD62P, CD63, activated GPIIb/IIIa, GPVI, GPIbα and formation platelet-leukocyte aggregates (PLA) were analyzed at rest and in response to stimulation. Delta granule storage-pool deficiency and secretion defects were also investigated. Fifteen healthy volunteers and ten patients with coronary artery disease served as controls. Results were also compared between survivors and non-survivors. Compared to controls, expression of platelet surface markers, delta granule secretion and formation of PLA was reduced, particularly in response to stimulation. Baseline CD63 expression was higher and activated GPIIb/IIIa expression in response to stimulation was lower in non-survivors on day 1 of ECMO. Logistic regression analysis revealed that these markers were associated with mortality. In conclusion, platelets from ECMO patients are severely dysfunctional predisposing patients to bleeding complications and poor outcome. Platelet dysfunction on day 1 of ECMO detected by the platelet surface markers CD63 and activated GPIIb/IIIa is associated with mortality. CD63 and activated GPIIb/IIIa may therefore serve as novel prognostic biomarkers, but future studies are required to determine their true potential.

**Supplementary Information:**

The online version contains supplementary material available at 10.1007/s11239-021-02562-9.

## Introduction

Extracorporeal membrane oxygenation (ECMO) is being increasingly used for critically ill patients requiring hemodynamic or respiratory support [1]. Veno-venous (VV-) ECMO is used for patients with respiratory failure and has gained importance during the ongoing COVID-19 pandemic [2]. Veno-arterial (VA-) ECMO is used in cardiac failure (e.g., protracted cardiogenic shock) and during cardiopulmonary resuscitation (eCPR) [3, 4].

Mortality is high in ECMO patients [5] and specific ECMO-related complications, for example bleeding and thrombotic complications contribute to the poor outcome [6, 7]. Recent investigations demonstrated that platelet dysfunction is an important part of the ECMO-associated coagulopathy, but many parameters of platelet function and their relation to clinical outcome in this patient group remain to be investigated [8].

For example, platelet surface expression of P-selectin (CD62P), CD63 and the activated conformation of GPIIb/IIIa (detected via the activation-specific antibody PAC-1) and changes in response to stimulants like adenosine diphosphate (ADP) and thrombin receptor activating peptide (TRAP) can be assessed using flow cytometry [9]. Furthermore, platelets can be stained with the fluorescent dye mepacrine, which is rapidly taken up and stored in delta granules. Granule release occurs in response to thrombin stimulation and can be monitored by flow cytometry in real-time allowing the diagnosis of acquired delta granule release defects [10, 11].

Moreover, activated platelets have been shown to interact with leukocytes forming platelet leukocyte aggregates (PLA) which are known mediators of vascular inflammation. PLA have also been advocated as more sensitive markers of platelet activation compared to CD62P or CD63 [12]. Also, the surface markers GPVI and GPIbα should be analyzed as they mediate important platelet functions required for primary hemostasis [9].

The aim of this study was to investigate platelet function in patients receiving ECMO, compare it with healthy controls and patients with coronary artery disease and to assess the association with outcome.

## Methods

Methodology regarding ECMO management and flow cytometric analysis is provided in the Supplementary Methods S1.

### Patient recruitment and blood sampling

The hospital patient data management system was screened to identify patients receiving VV- or VA-ECMO in the medical and heart surgical intensive care units of the University Hospital Freiburg, Germany, from December 2019 until January 2021. Study participants were 18 years or older with a hemoglobin- (Hb-) value over 8 g/dl. Patients with hematological malignancies were excluded.

Citrated blood (containing 3.2 % tri-sodium citrate, Sarstedt, Germany) was slowly drawn from an arterial line on day 1 (6–24 h after ECMO initiation), day 3 (72 ± 12 h after ECMO initiation) and after ECMO explantation (‘Post’, < 12 h after ECMO explantation).

Patients with coronary artery disease (CAD) were recruited 5–8 h after coronary angiography, which confirmed stable coronary heart disease. No stents were placed during coronary angiography. CAD patients had hemoglobin levels over 8 g/dl and no hematological malignancies. Citrated blood was drawn by antecubital vein puncture and transported to the laboratory immediately.

Clinical and laboratory parameters from ECMO patients and CAD patients were obtained from the electronic patient data management system closest to the time point of blood sampling.

Major bleeding was defined as previously described [13]. Major bleeding in postcardiotomy shock ECMO patients was only counted if it occurred 12 h after ECMO implantation and was not directly related to surgical intervention.

Healthy volunteers (healthy) were over 18 years, free of disease and had not taken any medication in the past 14 days. Blood was taken by antecubital vein puncture.

### Statistics

Variables are presented as mean ± SEM unless indicated otherwise. Continuous clinical variables (e.g., age, laboratory values) are presented as median (interquartile range). To account for repeated measures per patient, mixed effects models which allow missing data were used to compare differences of means across different time points in ECMO patients. One-way ANOVA followed by Tukey’s post hoc analysis was used to analyze differences of means of three or more unpaired variables. Unpaired Student’s t-tests were used to compare means of two unpaired variables. Simple logistic regression analysis was performed to determine association of platelet function parameters on day 1 with mortality. Areas under the receiver operating characteristic (ROC) curve were calculated to determine predictive accuracy of these parameters. A *p*-value ≤ 0.05 was considered statistically significant. Analysis was performed using GraphPad Prism V.9.0.2 (GraphPad Software, San Diego, California, USA).

## Results

### Clinical characteristics

Thirty patients receiving ECMO were recruited from December 2019 until January 2021. Clinical characteristics are presented in Table 1. Thirty patients were included on day 1, of which seven were female. Due to early death or early ECMO explantation, 19 of these patients were followed up on day 3 and 17 patients after explantation. Median patient age was 61 years. Twenty patients received VA-ECMO, and 10 patients received VV-ECMO. Main indications for VA-ECMO were cardiogenic shock or eCPR. Of the patients requiring VA-ECMO, 9 patients received VA-ECMO for severe postcardiotomy shock. The main indication for VV-ECMO was acute respiratory distress syndrome (ARDS). Median time on ECMO was 5 days. Fifteen patients survived until discharge from the intensive care wards and were counted as survivors. To reduce the influence of the underlying disease and cannulation technique on platelet function in the ECMO group, platelet function was also analyzed in a more homogeneous subgroup composed of the 12 VA-ECMO patients with known coronary artery disease. Clinical characteristics of this subgroup are presented in Supplementary Table S1.

**Table 1:** Clinical characteristics of ECMO patients. The results are presented as median (interquartile range, Q1-Q3) for continuous variables, and number (percentage) for categorical variables. Denominator of the percentage is the total number of subjects in the group. Laboratory parameters and ventilation settings presented were taken from the patient data management system on day 1 closest to the time of blood sampling for platelet function analysis. ALT, alanine aminotransferase; ARDS, acute respiratory distress syndrome; AST, aspartate aminotransferase; CRP, C-reactive protein; eCPR, extracorporeal cardiopulmonary resuscitation; F_i_O, fraction of inspired oxygen; INR, international normalized ratio; LDH, lactate dehydrogenase; VA-ECMO, veno-arterial extracorporeal membrane oxygenation; VV-ECMO, veno-venous extracorporeal membrane oxygenation. p_a_O_2_, partial oxygen pressure in arterial blood; p_a_CO_2,_ partial pressure of carbon dioxide in arterial blood; PEEP, positive endexpiratory pressure; PTT, partial thromboplastin time; SOFA, sequential organ failure assessment; WBC, white blood cells

Parameter	ECMO patients
Patients, n (%)	30 (100)
Age, y (Q1-Q3)	61 (50–71)
Female, n (%)	7 (23)
Survivors, n (%)	15 (50)
VV-ECMO, n (%)	10 (33)
VA-ECMO, n (%)	20 (67)
ECMO system, n (%)	
Stöckert Sorin	12 (40)
Maquet	13 (43)
Deltastream	4 (13)
CARL	1 (3)
Days on ECMO (median, Q1-Q3)	5.0 (3.0–7.0)
ECMO blood flow on day 1 (l/min, Q1-Q3)	4.2 (3.3–4.9)
Indications for VA-ECMO, n (%)	
Cardiogenic shock	17 (57)
- Postoperative	9 (30)
- Myocardial Infarction	2 (7)
- Cardiomyopathy	1 (3)
- Post cardiac arrest	3 (10)
- Endocarditis	1 (3)
- Pulmonary Embolism	1 (3)
eCPR	3 (10)
Indications for VV-ECMO, n (%)	
ARDS	9 (30)
- Primary	7 (23)
- Secondary	2 (7)
Pulmonary Embolism	1 (3)
Coronary artery disease, n (%)	12 (40)
Severe valvular heart disease, n (%)	5 (17)
Atrial fibrillation, n (%)	7 (23)
Diabetes mellitus, n (%)	3 (10)
Hypertension, n (%)	2 (7)
Smoking, n (%)	3 (10)
Hypercholesterolemia, n (%)	4 (13)
Cancer, n (%)	1 (3)
Acute renal failure during ECMO, n (%)	13 (43)
Acute liver failure during ECMO, n (%)	3 (10)
Major bleeding during ECMO, n (%)	15 (50)
Thrombotic events during ECMO, n (%)	3 (10)
Received Heparin during ECMO, n (%)	26 (87)
Received ASA during ECMO, n (%)	15 (50)
Received ASA + P_2_Y_12_ inhibitor during ECMO, n (%)	7 (23)
Received transfusions during ECMO, n (%)	18 (60)
Mechanical ventilation on day 1, n (%)	30 (100)
SOFA score on day 1 (Q1-Q3)	11 (10–13)
WBC (x10^3^ /µl, Q1-Q3)	10.2 (6.1–17.6)
Platelets (x10^3^ /µl, Q1-Q3)	112 (61.0-184.3)
Hemoglobin (g/dl, Q1-Q3)	8.8 (8.1–10.5)
Creatinine (mg/dl, Q1-Q3)	1.7 (0.9–2.5)
Urea (mg/dl, Q1-Q3)	54.0 (37.5–73.5)
Bilirubin (mg/dl, Q1-Q3)	1.9 (1.2–3.5)
AST (U/l, Q1-Q3)	161 (76.3-385.5)
ALT (U/l, Q1-Q3)	62.5 (35.3-113.8)
CRP (mg/l, Q1-Q3)	104.1 (29.3-196.7)
LDH (U/l)	473 (396.0-1210.0)
PTT (s, Q1-Q3)	45.5 (38.0-64.2)
INR (Q1-Q3)	1.2 (1.1–1.4)
Fibrinogen (mg/dl)	275.0 (158.0-508.0)
Lactate (mmol/l, Q1-Q3)	2.6 (1.4–3.9)
p_a_O_2_ (mmHg, Q1-Q3)	105.5 (73.1-159.3)
p_a_CO_2_ (mmHg, Q1-Q3)	40.7 (34.8–42.9)
F_i_O (%, Q1-Q3)	50.0 (40.0–50.0)
PEEP (mbar, Q1-Q3)	10.5 (7.0–15.0)
Respiratory rate (/min, Q1-Q3)	16.0 (12.0-18.25)

Ten control patients with stable coronary artery disease were recruited with a median age of 67 years. Four CAD patients were female. Clinical characteristics of CAD patients can be found in Supplementary Table S2. Fifteen healthy controls with a median age of 28 years were recruited. Eight were female, 7 were male.

### Platelet count in ECMO patients is lower than in CAD patients

Platelet counts in ECMO patients decreased during time on ECMO but showed a rising tendency after ECMO explantation (Platelet count x 10^3^/µl day 1 vs. day 3 vs. post: 129 ± 17 vs. 86 ± 9 vs. 110 ± 13, p = 0.04 day 1 vs. day 3, Supplementary Figure S1). Compared to CAD patients (Platelet count x 10^3^/µl: 243 ± 17), platelet counts during ECMO and after ECMO explantation were significantly lower (p < 0.001). In the subgroup of VA-ECMO patients, platelet counts were also significantly lower compared to CAD patients (Supplementary Figure S2).

### Mepacrine assay reveals delta granule secretion defects in ECMO patients

To further assess delta granule secretion defects in ECMO patients a mepacrine assay was conducted. Thrombin stimulation of mepacrine stained platelets led to a significant reduction in mean fluorescence intensity compared to PBS treatment in ECMO patients at all time points, healthy controls and CAD patients indicating that the established assay worked well (Supplementary Figure S3). After 20 min of thrombin stimulation mepacrine release was significantly lower in ECMO patients compared to controls (Supplementary Figure S4) indicating a reduced capacity to secrete delta granules (e.g., percentage of baseline mean fluorescence intensity (MFI): day 1 vs. CAD vs. healthy: 79.7 ± 1.9 vs. 70.6 ± 2.8 vs. 63.2 ± 2.1, p = 0.031 vs. CAD, p < 0.001 vs. healthy). This characteristic was also present after ECMO explantation (Percentage of baseline MFI - post: 80.3 ± 2.5, p = 0.03 vs. CAD, p < 0.001 vs. healthy controls).

### Platelet CD62P and CD63 expression differs significantly from controls

Common markers of platelet activation (CD62P, CD63) at baseline (unstimulated samples) and in response to stimulation were investigated in ECMO patients at different time points and compared with controls.

**Fig. 1:**
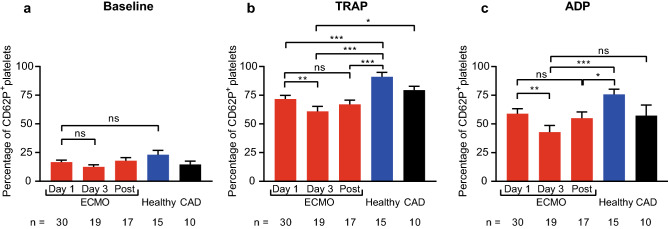
Platelet expression of CD62P in patients receiving extracorporeal membrane oxygenation (ECMO) compared to controls. CD62P expression was analyzed by flow cytometry on resting (baseline), thrombin receptor activating peptide (TRAP)-stimulated and adenosine diphosphate (ADP)-stimulated platelets. Blood was sampled from ECMO patients on day1, day 3 and after ECMO explantation (Post). CD62P expression on platelets was compared to healthy controls (Healthy) and patients with coronary artery disease (CAD). **a**, Baseline CD62P expression was similar in ECMO patients and controls. CD62P expression in response to TRAP (**b**) and ADP (**c**) stimulation was lower in ECMO patients. The number of ECMO patients remaining at each time point and the number of control patients are indicated below. Data are presented as mean ± standard error of the mean. ns, not significant, *p < 0.05, **p < 0.01, ***p < 0.001

Baseline platelet CD62P expression (Fig. 1) did not significantly differ between ECMO patients and controls and did not change at different time points. However, CD62P expression in response to ADP and TRAP stimulation was significantly lower on day 3 compared to day 1 (CD62P expression in percentage day 1 vs. day 3: TRAP: 71.7 ± 3.2 vs. 61.0 ± 4.3, p = 0.007, ADP: 59.0 ± 4.3 vs. 42.9 ± 5.8, p = 0.001). Moreover, CD62P expression in response to stimulation with TRAP and ADP in ECMO patients was significantly reduced compared to healthy controls (CD62P expression in percentage day 3 vs. healthy controls: TRAP: 61.0 ± 4.3 vs. 91.0 ± 3.8, p < 0.001, ADP: 42.9 ± 5.8 vs. 75.8 ± 4.5, p < 0.001). Compared to CAD patients, ECMO patients on day 3 also showed a significantly reduced CD62P expression in response to TRAP (CD62P expression in percentage day 3 vs. CAD, TRAP: 61.0 ± 4.3 vs. 79.5 ± 3.3, p = 0.02), but not ADP stimulation. Platelet CD62P expression in response to stimulation was also significantly lower in the VA-ECMO subgroup compared to controls (Supplementary Figure S5).

**Fig. 2:**
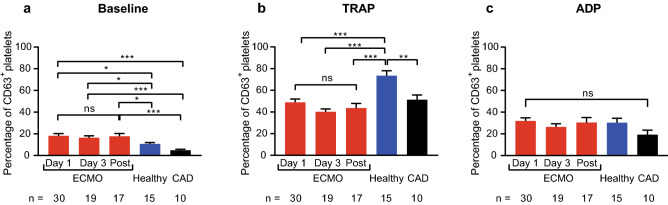
Platelet expression of CD63 in patients receiving extracorporeal membrane oxygenation (ECMO) compared to controls. CD63 expression was analyzed by flow cytometry on resting (baseline), thrombin receptor activating peptide (TRAP)-stimulated and adenosine diphosphate (ADP)-stimulated platelets. Blood was sampled from ECMO patients (ECMO) on day 1, day 3 and after ECMO explantation (Post). CD63 expression on platelets was compared to healthy controls (Healthy) and patients with coronary artery disease (CAD). **a**, Baseline CD63 expression was significantly elevated on platelets from ECMO patients compared to controls. **b**, CD63 expression in response to TRAP was lower in ECMO patients compared to healthy controls. **c**, CD63 expression in response to ADP stimulation was similar in ECMO patients and controls. The number of ECMO patients remaining at each time point and the number of control patients are indicated below. Data are presented as mean ± standard error of the mean. ns, not significant, *p < 0.05, **p < 0.01, ***p < 0.001

CD63 expression at baseline and in response to stimulation did not change significantly in ECMO patients at different time points (Fig. 2). In contrast to CD62P however, baseline CD63 expression was significantly higher in ECMO patients at all time points compared to controls (e.g., CD63 expression in percentage day 1 vs. healthy vs. CAD: 18.2 ± 2.0 vs. 10.7 ± 1.3 vs. 4.8 ± 0.9, p = 0.02 vs. healthy, p < 0.001 vs. CAD). Response to stimulation with TRAP and ADP was also different from CD62P. Whereas significantly reduced CD63 expression in response to TRAP stimulation at all time points was found compared to healthy controls (e.g., CD63 expression in percentage day 1 vs. healthy: 48.9 ± 3.1 vs. 73.5 ± 4.6, p < 0.001), platelet CD63 expression in ECMO patients in response to TRAP was not different from CAD patients. Furthermore, CD63 expression in response to ADP did not significantly differ between ECMO patients, healthy controls, and CAD patients. In the VA-ECMO subgroup baseline CD63 expression was also increased compared to CAD patients. Furthermore, CD63 expression in response to TRAP stimulation was also significantly lower compared to healthy controls (Supplementary Figure S6).

**Expression of activated GPIIb/IIIa on platelets in response to platelet stimulants is reduced in ECMO patients compared to controls**.

**Fig. 3:**
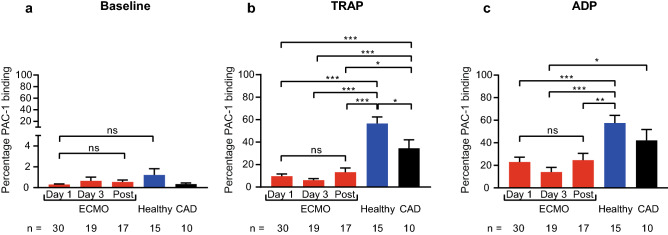
Expression of activated GPIIb/IIIa in patients receiving extracorporeal membrane oxygenation (ECMO) compared to controls. Expression of activated GPIIb/IIIa was analyzed by flow cytometry on resting (baseline), thrombin receptor activating peptide (TRAP)-stimulated and adenosine diphosphate (ADP)-stimulated platelets using the conformation specific antibody PAC-1. Blood was sampled from ECMO patients (ECMO) on day 1, day 3 and after ECMO explantation (Post). Expression of activated GPIIb/IIIa on platelets was compared to healthy controls (Healthy) and patients with coronary artery disease (CAD). **a**, Baseline expression of activated GPIIb/IIIa did not significantly differ between ECMO patients or controls. **b**, Expression of activated GPIIb/IIIa in response to TRAP stimulation was significantly lower in ECMO patients at all time points compared to healthy controls and CAD patients. **c**, Expression of activated GPIIb/IIIa in response to ADP stimulation in ECMO patients was also significantly lower compared to controls. The number of ECMO patients remaining at each time point and the number of control patients are indicated below. Data are presented as mean ± standard error of the mean. ns, not significant, *p < 0.05, **p < 0.01, ***p < 0.001

Using the activation-specific anti-GPIIb/IIIa antibody PAC-1, we determined GPIIb/IIIa activation status on platelets from ECMO patients at baseline and in response to ADP and TRAP stimulation (Fig. 3). Baseline expression of activated GPIIb/IIIa was low, did not change at different time points in ECMO patients and did not differ significantly from controls. However, expression of activated GPIIb/IIIa in response to TRAP stimulation was significantly lower in ECMO patients at all time points compared to controls (e.g., percentage PAC-1 binding day 3 vs. healthy vs. CAD: 6.0 ± 1.5 vs. 56.5 ± 5.8 vs. 34.6 ± 7.5, p < 0.001). Similar observations were made in response to ADP stimulation (e.g., percentage PAC-1 binding day 3 vs. healthy vs. CAD: 14.0 ± 4.2 vs. 57.5 ± 6.8 vs. 42.2 ± 9.6, p < 0.001 day 3 vs. healthy, p = 0.01 vs. CAD). Expression of activated GPIIb/IIIa in response to stimulation in the VA-ECMO subgroup was also lower compared to controls (Supplementary Figure S7).

### Platelets from ECMO patients have lower levels of GPVI and GPIbα on their surface

To detect potential receptor shedding of GPVI and GPIbα in ECMO patients, expression of the parameters on platelets were analyzed (Supplementary Figure S8). GPVI and GPIbα expression on platelets from ECMO patients did not change significantly at different time points. However, compared to healthy controls and CAD patients, GPVI expression levels were significantly lower in ECMO patients (e.g., MFI day 1 vs. healthy vs. CAD: 2,261 ± 95.8 vs. 3,115 ± 182.5 vs. 3,481 ± 87.5, p < 0.001). Differences in GPIbα expression on platelets from ECMO compared to controls were less pronounced, but significance was achieved compared to CAD patients (MFI day 1 vs. CAD: 12,171 ± 641.8 vs. 15,349 ± 589.5, p = 0.03). GPVI expression in the VA-ECMO subgroup was also significantly lower compared to controls, whereas no significant differences in GPIbα expression between the VA-ECMO subgroup and controls were detected (Supplementary Figure S9).

### Formation of platelet leukocyte aggregates in response to stimulation is reduced in ECMO patients

**Fig. 4:**
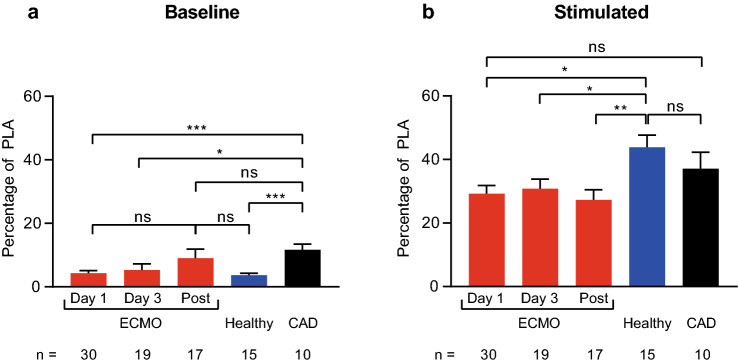
Levels of platelet leukocyte aggregates (PLA) in patients receiving extracorporeal membrane oxygenation (ECMO) compared to controls. The percentage of CD61^+^/CD45^+^ PLA of all CD45^+^ leukocytes is presented. PLA were analyzed at rest (‘baseline’) and in response to simulation with adenosine diphosphate (ADP) and phorbol 12-myristate 13-acetate (‘stimulated’). Blood was sampled from ECMO patients (ECMO) on day 1, day 3 and after ECMO explantation (Post). PLA in ECMO patients were compared to healthy controls (Healthy) and patients with coronary artery disease (CAD). **a**, baseline PLA levels were similar in ECMO patients and healthy controls. CAD patients had higher levels of PLA. **b**, PLA formation in response to stimulation was significantly reduced in ECMO patients and reached significance at all time points compared to healthy controls. The number of ECMO patients remaining at each time point and the number of control patients are indicated below. Data are presented as mean ± standard error of the mean. ns, not significant, *p < 0.05, **p < 0.01, ***p < 0.001

Since PLA are known mediators of vascular inflammation [12], we investigated their levels in ECMO patients (Fig. 4). PLA levels at baseline and in response to stimulation in all ECMO patients did not change significantly during ECMO and after explantation. However, baseline PLA levels were significantly lower compared to CAD patients, but not healthy controls (day 1 vs. healthy vs. CAD: 4.3 ± 0.8 vs. 3.7 ± 0.6 vs. 11.7 ± 1.8, p < 0.001 day 1 and healthy vs. CAD). PLA levels in response to stimulation with ADP + PMA were significantly lower compared to healthy controls, but not CAD patients (day 1 vs. healthy vs. CAD: 29.2 ± 2.6 vs. 43.8 ± 3.9 vs. 37.1 ± 5.1, p = 0.01 day 1 vs. healthy). PLA levels in the VA-ECMO subgroup showed similar differences compared to controls as reported for all ECMO patients (Supplementary Figure S10).

### Levels of activated GPIIb/IIIa and CD63 on day 1 of ECMO are associated with mortality

Next, we investigated whether parameters of platelet function were differentially expressed in survivors and non-survivors on day 1 of ECMO and associated with mortality. An overview of platelet function parameters in survivors and non-survivors on day 1 of ECMO can be found in Table 2. Clinical characteristics of survivors and non-survivors are shown in Supplementary Table S3. Baseline CD63 expression was significantly higher in non-survivors (p = 0.003). The area under the ROC curve for mortality was 0.79 (p = 0.007). Furthermore, expression of activated GPIIb/IIIa in response to TRAP and ADP stimulation was significantly lower in non-survivors (p = 0.009). The area under the ROC curve for mortality was 0.73 for TRAP stimulation (p = 0.03) and 0.75 for ADP stimulation (p = 0.02).

**Table 2:** Platelet function parameters in survivors and non-survivors on day 1 of ECMO. Data are presented as mean ± SEM, n = 15 survivors, n = 15 non-survivors; p-values were calculated by an unpaired Student’s t-test. p-values < 0.05 are highlighted in bold. Parameters were recorded as described in the Methods section. ECMO, extracorporeal membrane oxygenation; MFI, mean fluorescence intensity; PLA, platelet leukocyte aggregates

Parameter	Survivors	Non-Survivors	p-value
Baseline CD62P expression (%)	16.7 ± 2.4	16.6 ± 2.7	> 0.99
CD62P + TRAP (%)	74.9 ± 3.9	68.5 ± 4.9	0.32
CD62P + ADP (%)	66.7 ± 6.3	51.3 ± 5.5	0.07
Baseline CD63 expression (%)	12.4 ± 2.0	24.0 ± 2.8	**0.003**
CD63 + TRAP (%)	47.3 ± 5.4	50.4 ± 3.2	0.63
CD63 + ADP (%)	31.1 ± 4.6	32.8 ± 3.4	0.77
Baseline PAC-1 binding (%)	0.34 ± 0.08	0.27 ± 0.06	0.47
PAC-1 + TRAP (%)	13.8 ± 3.3	5.4 ± 2.1	**0.04**
PAC-1 + ADP (%)	33.7 ± 6.5	12.3 ± 4.0	**0.009**
GPVI (MFI)	2,271.0 ± 115.6	2,248.0 ± 162.0	0.91
GPIbα (MFI)	11,440 ± 797.9	12,955 ± 1,006	0.25
Baseline PLA (%)	2.9 ± 0.8	5.8 ± 1.4	0.07
Stimulated PLA (%)	30.4 ± 3.6	28.0 ± 4.0	0.66
Mepacrine Assay - % of baseline MFI after 20 min thrombin stimulation	78.1 ± 2.7	81.4 ± 2.7	0.39
Platelet count x10^3^/µl	142.2 ± 15.8	116.5 ± 30.6	0.46

## Discussion

This study reveals multiple signs of platelet dysfunction in ECMO patients and indicates that platelet dysfunction on day 1 of ECMO as determined by CD63 or activated GPIIb/IIIa is associated with mortality. To the best of our knowledge, this study is the first to establish a link between platelet dysfunction and mortality in patients receiving ECMO.

Compared to healthy controls, significantly reduced expression of platelet surface markers CD62P and CD63 in response to stimulation was shown which is in line with previous reports. A recent study suggested that platelets from patients receiving ECMO might therefore be in an “exhausted state” resulting in an increased risk of bleeding complications [8, 14, 15].

However, previous studies investigating platelet function in ECMO patients often recruited healthy controls [14, 15], which differ from ECMO patients in several regards. In this study, CAD patients, which may share several comorbidities with ECMO patients, served as stricter controls in an aim to put platelet dysfunction in ECMO patients into perspective. As expected, differences in the expression of platelet surface markers in response to stimulation compared to CAD patients were not as strong as compared to healthy controls. This can be explained by the fact that 6 out of 10 CAD patients received P_2_Y_12_ inhibitors and 7/10 received ASA. In previous studies, the reduced platelet response to stimulation compared to *healthy controls* may therefore have been overemphasized regarding its *clinical relevance*. For instance, CAD patients receive dual anti-platelet therapy after coronary stenting for 6–12 months and major bleeding is reported in only approximately 1.7 % on average [16] which stands in contrast to a 50 % [17] bleeding rate for ECMO patients, which was also observed in our cohort.

Therefore, markers of platelet function which are related to patient outcome may be advantageous for assessing platelet dysfunction in ECMO patients. For instance, *baseline* CD63 in ECMO patients is a highly promising marker. Although elevated levels of CD63 on platelets from patients with *ventricular assist devices* have been reported [18], to the best of our knowledge, this study is the first to report significantly elevated baseline CD63 expression on platelets from *ECMO* patients compared to healthy controls and CAD patients. Interestingly, higher baseline levels of CD63 on day 1 of ECMO were associated with increased mortality.

Furthermore, we are the first group to demonstrate a significantly reduced mepacrine release in response to thrombin stimulation in ECMO patients compared to healthy controls and CAD patients [19]. Since CD63 is contained in delta granules and translocated to the surface upon platelet activation, a combination of elevated baseline CD63 expression and a reduced mepacrine release as reported in this study could indicate acquired delta granule secretion defects in ECMO patients caused by platelet activation and degranulation in the extracorporeal circuit [20, 21]. This phenomenon could be another factor contributing to an increased risk of bleeding in ECMO patients.

In this study, a reduced capacity of platelets to express activated GPIIb/IIIa in response to stimulation was demonstrated which may be caused by receptor shedding due to pathological shear stress in the extracorporeal circuit [22]. Activated GPIIb/IIIa is required for platelet aggregation [23] and dysfunction of GPIIb/IIIa has previously been associated with an increased bleeding risk in uremic patients [24] or patients treated with GPIIb/IIIa receptor inhibitors [25]. Therefore, a reduced capacity of platelets to express this receptor could be predisposing ECMO patients to severe bleeding complications. Moreover, our data provides the underlying mechanism behind previous reports of reduced platelet aggregation in ECMO patients [14, 26]. Mazzeffi et al. recently reported reduced expression of activated GPIIb/IIIa in response to stimulation on platelets from patients receiving VA-ECMO, but the study lacked a control group and included only 10 ECMO patients. Furthermore, no relation to outcome was described [27]. In contrast, our study demonstrates that low levels of activated GPIIb/IIIa in response to stimulation on day 1 of ECMO were associated with mortality.

Receptor shedding in the extracorporeal circuit has been reported for the platelet surface markers GPVI and GPIbα [28]. Reduced levels of GPVI compared to healthy controls and CAD patients were also observed in our study. Furthermore, GPIbα was significantly reduced compared to CAD patients. Loss of these platelet adhesion receptors is expected to contribute to the increased risk of bleeding in ECMO patients [28].

Furthermore, platelet count in ECMO patients was significantly reduced compared to CAD patients. Thrombocytopenia in ECMO patients is in line with previous reports [29] and has been suggested to contribute to bleeding complications [6]. Causes for thrombocytopenia include the underlying disease (e.g., sepsis), platelet activation, adhesion, and loss in the extracorporeal circuit and hemodilution [30]. The risk of major bleeding for ECMO patients carried by thrombocytopenia alone may be significantly increased by the severe signs of platelet dysfunction documented in our study [31].

Interestingly, for most platelet function parameters investigated, signs of platelet dysfunction persisted even after explantation of ECMO suggesting platelets remain in a dysfunctional state for some time after ECMO removal. Clinicians should be aware that even after weaning off ECMO, patients could still carry a significant risk of bleeding.

Platelets are emerging as important immune cells with distinct functions [32], for example in sepsis where they play an important role in alerting leukocytes and mediating bacterial clearance [33]. Platelets readily form aggregates with leukocytes which have also been shown to mediate important immune functions, such as facilitation of leukocyte extravasation to the site of inflammation [34] and release of neutrophil extracellular traps [35]. To the best of our knowledge, this study is the first to demonstrate a reduced formation of PLA in ECMO patients. This finding could indicate that platelets are not capable of exerting some of their immunological functions which is in line with recent reports of *immunoparalysis* in ECMO patients associated with infection and poor outcome [36–39]. Furthermore, the association of platelet dysfunction with mortality may therefore not only be due to increased bleeding complications but also involve immunological mechanisms.

Our study is not without limitations. Due to the observational nature of the study, we cannot report a *causal* connection between ECMO therapy and platelet dysfunction. The association between platelet dysfunction and initiation of ECMO therapy may have been substantiated by adding a pre-ECMO time point, which was not featured in this study. Moreover, platelet function in ECMO patients may be influenced by a range of different factors, including the underlying disease, the medication, and the cannulation strategy (VA- vs. VV-ECMO). However, due to the relatively small sample size, the possibility of multivariate and subgroup analysis was limited.

In an effort to reduce the influence of the underlying disease and cannulation technique on platelet function in the ECMO group, platelet function was also analyzed in a more homogeneous subgroup composed of the 12 VA-ECMO patients with known coronary artery disease. Interestingly, most results indicating platelet dysfunction in the whole ECMO group, e.g., the reduced response to stimulation compared to controls, were also found in the subgroup of VA-ECMO patients with known coronary artery disease. This could indicate that that ECMO therapy exerts a much stronger influence on platelet function than other parameters, such as medication and the underlying disease and supports our strategy of recruiting both VV- and VA-ECMO patients.

In conclusion, platelets from ECMO patients are severely dysfunctional predisposing patients to bleeding complications and poor outcome. Platelet dysfunction on day 1 of ECMO detected by the platelet surface markers CD63 and activated GPIIb/IIIa is associated with mortality. CD63 and activated GPIIb/IIIa may therefore serve as novel prognostic biomarkers, but future studies are required to determine their true potential.

## Supplementary Information

Below is the link to the electronic supplementary material.

Supplementary material 1 (PDF 196.9 kb)

Supplementary material 2 (PDF 1423.1 kb)

Supplementary material 3 (PDF 229.0 kb)

## Data Availability

The datasets used and/or analysed during the current study are available from the corresponding author on reasonable request.
