# Supplementary material for: Early platelet dysfunction in patients receiving extracorporeal membrane oxygenation is associated with mortality
Source: J Thromb Thrombolysis. 2021 Sep 16;53(3):712–21. doi: 10.1007/s11239-021-02562-9 (PMC8444511; doi:10.1007/s11239-021-02562-9)
Supplement: Supplementary file 1 — Supplementary material 1 (PDF 196.9 kb) [file 11239_2021_2562_MOESM1_ESM.pdf]

Early platelet dysfunction in patients receiving extracorporeal membrane oxygenation is associated with mortality

Journal of Thrombosis and Thrombolysis

Patrick M. Siegel, MD[1]; Julia Chalupsky, M.Sc.[1]; Christoph B. Olivier, MD[1]; István Bojti, MD[1]; Jan-Steffen Pooth, MD[2]; Georg Trummer, MD[2]; Christoph Bode, MD[1]; Philipp Diehl, MD, PhD[1]

[1] Department of Cardiology and Angiology I, University Heart Center Freiburg – Bad Krozingen, Faculty of Medicine, University of Freiburg, Freiburg, Germany

[2] Department of Cardiovascular Surgery, University Heart Center Freiburg – Bad Krozingen, Faculty of Medicine, University of Freiburg, Freiburg i. Br., Germany

Corresponding author

Dr. Patrick Malcolm Siegel

Department of Cardiology and Angiology I, Heart Center Freiburg University

Faculty of Medicine, University of Freiburg

Freiburg, 79106

Germany

Tel: +49 761/270-34010

E-Mail: patrick.siegel@universitaets-herzzentrum.de

Supplementary Methods S1

ECMO indications and management

Decision on ECMO placement was made by an experienced ECMO physician who was part of a 24 h ECMO response team. Implantation and management were carried out as described previously [1,2]. Cannulation was performed in Seldinger technique without a primary surgical cut. For VA-ECMO, venous cannulas had a diameter of 21-23 F and arterial cannulas had a diameter of 15-17 F. Distal limb perfusion was added if necessary. For VV-ECMO, most patients had a dual-lumen cannula inserted into the right V. jugularis interna (27-31 F, Avalon, Maquet, Rastatt, Germany).

ECMO patients received red blood cell transfusions as indicated to maintain hemoglobin levels above 8 g/dl. In patients without signs of bleeding or thrombosis (including the ECMO circuit) unfractionated heparin was used for anticoagulation to achieve a partial thromboplastin time (PTT) of 40-50 s. A platelet count of 50,000 or higher was aimed for. If patients showed signs of bleeding or thrombosis, individual targets for the PTT and platelet counts were set.

The systems used for ECMO included the Maquet Cardiohelp System with an HLS Set Advanced (Maquet Cardiopulmonary GmbH, Rastatt, Germany), the Stöckert® centrifugal pump (LivaNova PLC, London, United Kingdom), the Deltastream system (Xenios AG, Heilbronn, Germany) and the CARL system (Resuscitec, Freiburg, Germany). All systems included one oxygenator.

Flow Cytometry

Antibodies: The following antibodies were used: FITC anti-CD41/61 (clone: PAC-1, Biolegend, USA), BV421 anti-CD62P (clone: AK4, Biolegend, USA), APC anti-CD61 (clone: VI-PL2, Biolegend, USA), PE anti-CD63 (clone: H5C6, Biolegend, USA), PE anti-GPVI (clone: HY101, BD, USA), FITC anti-GPIbα (clone: AK2, Bio-Rad, USA), PE anti-CD45 (clone: HI30, Biolegend, USA).

Whole blood static platelet activation assay, GPVI and GPIbα staining: Citrated blood was left to rest for 10 min at room temperature (RT) after arrival in the laboratory to reduce platelet pre-activation. 5 µl of antibodies pre-diluted with phosphate buffered saline (PBS) were added to FACS tubes before blood was added and stored in the dark. The following dilutions were used: FITC anti-CD41/61 (PAC1, 1:5), BV421 anti-CD62P (1:100), APC anti-CD61 (1:10), PE anti-CD63 (1:10). Separate tubes containing APC anti-CD61 (1:10), FITC anti-GPIbα (1:100) and PE anti-GPVI (1:10) were prepared. Corresponding isotype control antibody dilutions were used. 500 µl of blood was stimulated with ADP (F_c=20 µM, Sigma-Aldrich, USA) or TRAP (F_c=32 µM, Sigma-Aldrich, USA) or PBS as a negative control for 2 min at RT. 5 µl of blood were added to the FACS-tubes containing the antibodies for staining (20 min, RT, dark). 1 ml of ice cold CellFIX^{TM} solution (BD, USA) was then added per tube and samples were incubated for 30 min at 4°C in the dark.

After pre-gating platelets in the FSC/SSC plot according to their assumed location, platelets were then identified according to their CD61 expression. GPVI and GPIbα expression on CD61$^+$ platelets were recorded as the mean fluorescence intensity (MFI) in the PE or FITC channel. Expression of CD62P or CD63 on and binding of PAC-1 to CD61$^+$ platelets was recorded as percentage. A gate including the top 1 % of the population in the corresponding isotype control sample was set to define the positive population. The population shifting into this gate in unstimulated (=*baseline*) or stimulated stained samples was recorded as percentage. 10,000 CD61$^+$ platelets were recorded per sample.

Platelet leukocyte aggregates*:* Whole citrated blood was stimulated with phorbol 12-myristate 13-acetate (PMA, 200 nM) and ADP (F_c=20 µM) or the same volume of PBS as a negative control. 5 µl of blood were added to FACS tubes containing pre-diluted antibodies, either PE anti-CD45 (5 µl of 1:10) + APC anti-CD61 (5 µl of 1:10) or PE anti-CD45 + APC isotype control. The percentagel of CD61$^+$/CD45$^+$ PLAs is presented. To calculate this value, gating was performed for CD45$^+$ leukocytes and a gate including the top 1 % of the population in the APC isotype control sample was set to define the positive population. The percentage

of CD61+ cells shifting into this gate in unstimulated or stimulated stained samples was recorded as percentage. 5,000 CD45+ leukocytes were recorded per sample.

Mepacrine assay: A modified version of a previously described protocol was used [3]. Citrated whole blood was diluted 1:40 in Hank's balanced salt solution (HBSS, ThermoFisher, USA). 300 µl of diluted whole blood was stained with mepacrine (F_c=100 µM, 30 min, 37°C) to encourage mepacrine uptake by platelets followed by 2.5 µl of an APC anti-CD61 antibody (20min, RT, dark) per sample. 100 µl of this solution was transferred to a new FACS tube and diluted 1:20 with HBSS. Acquisition was started, the CD61+ platelet population was identified and the FITC fluorescence intensity was recorded continuously. After 2 min of acquisition, thrombin (F_c=0.4 U/ml) or PBS (unstimulated sample) were added, and recording was continued for 20 min. The FITC MFI over time was analyzed using the FlowJo Kinetics function. Due to different baseline MFI values in healthy controls, CAD and ECMO patients the percentage change from baseline after stimulation was calculated. The mean of the FITC MFI during the first two minutes of acquisition before thrombin was added was defined as baseline. All samples were read at low flow rate on a FACS Canto II flow cytometer (BD, USA). Analysis was performed using FlowJo V10.6.0 (FlowJo LLC, USA).

References

1. Rilinger J, Zotzmann V, Bemtgen X, Schumacher C, Biever PM, Duerschmied D, Kaier K, Stachon P, von zur Mühlen C, Zehender M, Bode C, Staudacher DL, Wengenmayer T (2020) Prone positioning in severe ARDS requiring extracorporeal membrane oxygenation. Critical Care 24 (1):397. doi:10.1186/s13054-020-03110-2

2. Staudacher DL, Gold W, Biever PM, Bode C, Wengenmayer T (2017) Early fluid resuscitation and volume therapy in venoarterial extracorporeal membrane oxygenation. J Crit Care 37:130-135. doi:10.1016/j.jcrc.2016.09.017

3. Wall JE, Buijs-Wilts M, Arnold JT, Wang W, White MM, Jennings LK, Jackson CW (1995) A flow cytometric assay using mepacrine for study of uptake and release of platelet dense granule contents. Br J Haematol 89 (2):380-385. doi:10.1111/j.1365-2141.1995.tb03315.x
