# Supplementary material for: Early platelet dysfunction in patients receiving extracorporeal membrane oxygenation is associated with mortality
Source: J Thromb Thrombolysis. 2021 Sep 16;53(3):712–21. doi: 10.1007/s11239-021-02562-9 (PMC8444511; doi:10.1007/s11239-021-02562-9)
Supplement: Supplementary file 2 — Supplementary material 2 (PDF 1423.1 kb) [file 11239_2021_2562_MOESM2_ESM.pdf]

Early platelet dysfunction in patients receiving extracorporeal membrane oxygenation is associated with mortality

Journal of Thrombosis and Thrombolysis

Patrick M. Siegel, MD[1]; Julia Chalupsky, M.Sc.[1]; Christoph B. Olivier, MD[1]; István Bojti, MD[1]; Jan-Steffen Pooth, MD[2]; Georg Trummer, MD[2]; Christoph Bode, MD[1]; Philipp Diehl, MD, PhD[1]

[1] Department of Cardiology and Angiology I, University Heart Center Freiburg – Bad Krozingen, Faculty of Medicine, University of Freiburg, Freiburg, Germany

[2] Department of Cardiovascular Surgery, University Heart Center Freiburg – Bad Krozingen, Faculty of Medicine, University of Freiburg, Freiburg i. Br., Germany

<u>Corresponding author</u>

Dr. Patrick Malcolm Siegel

Department of Cardiology and Angiology I, Heart Center Freiburg University

Faculty of Medicine, University of Freiburg

Freiburg, 79106

Germany

Tel: +49 761/270-34010

E-Mail: patrick.siegel@universitaets-herzzentrum.de

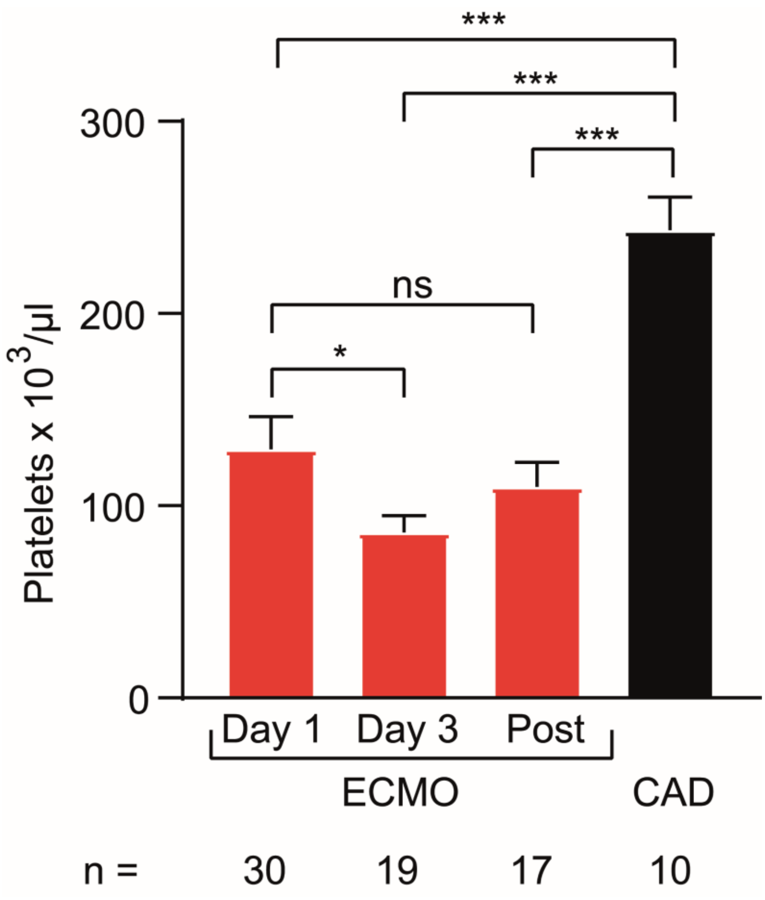

Supplementary Figure S1 Platelet count in extracorporeal membrane oxygenation (ECMO) patients compared to patients with stable coronary artery disease (CAD). Platelet counts decreased on day 3 compared to day 1 on ECMO and platelet counts in ECMO patients were significantly lower compared to CAD patients. Platelet counts were taken from the patient data management system. The number of ECMO patients remaining at each time point and the number of control patients are indicated below. Data are presented as mean±standard error of the mean. ns, not significant, *p<0.05, ***p<0.001

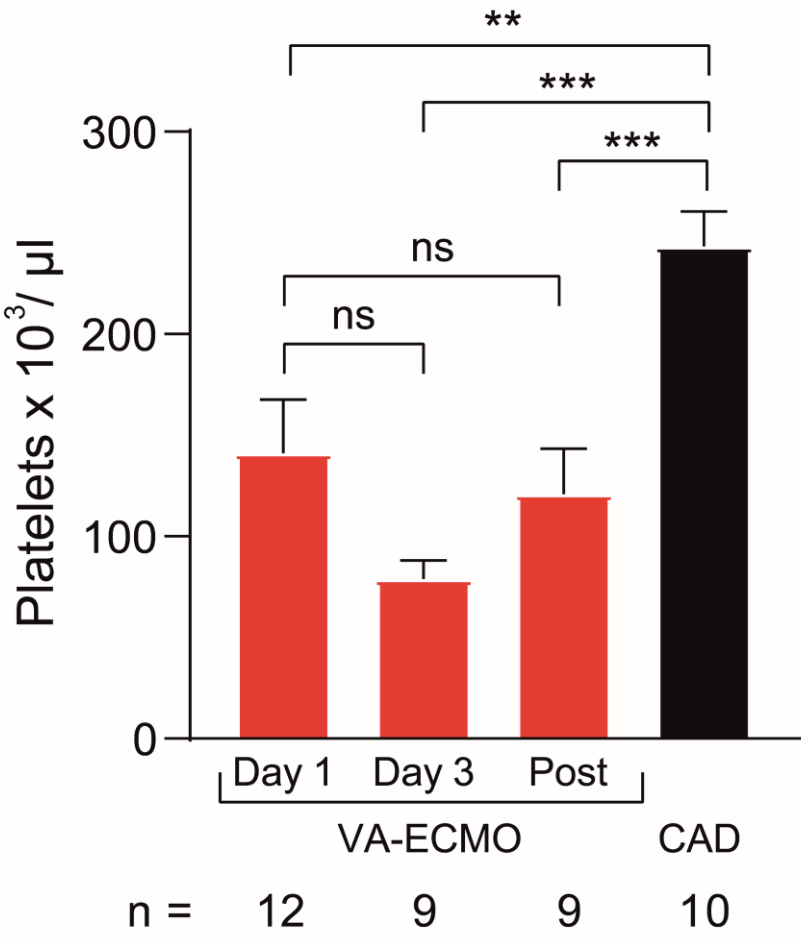

Supplementary Figure S2 Platelet count in patients receiving *veno-arterial extracorporeal membrane oxygenation (VA-ECMO)* compared to patients with stable coronary stable artery disease (CAD). Only VA-ECMO patients with known coronary artery disease are presented. The number of VA-ECMO patients remaining at each time point and the number of control patients are indicated below. Data are presented as mean±standard error of the mean. ns, not significant, $^{**}p<0.01$, $^{***}p<0.001$

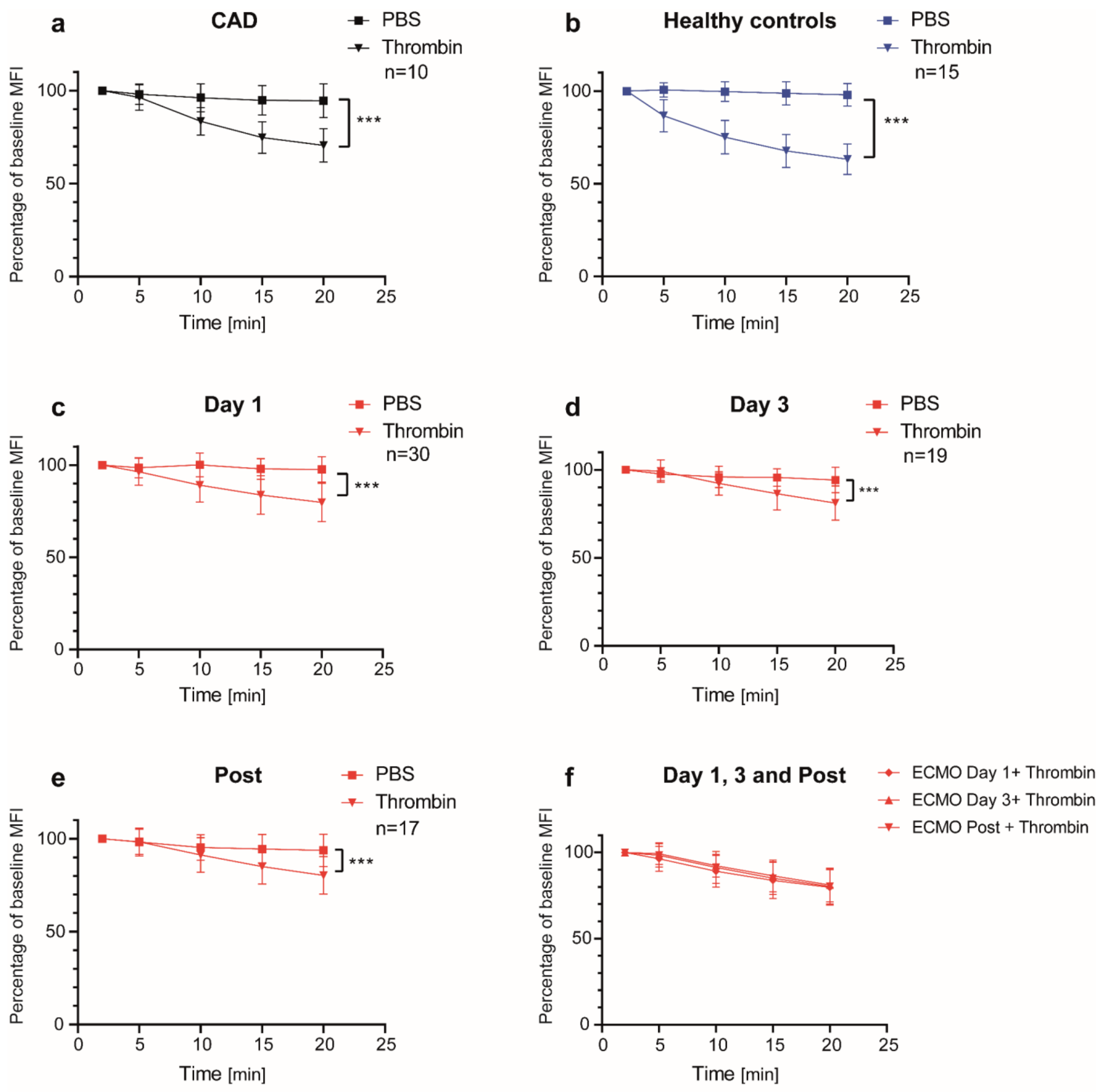

Supplementary Figure S3 Platelet mepacrine release after thrombin stimulation in comparison to PBS. The fluorescent dye mepacrine is taken up by platelets and stored in delta granules. Thrombin stimulation leads to release of delta granules, which can be quantified by a reduction in platelet mean fluorescence intensity (MFI). The following groups were investigated: Patients with coronary artery disease (CAD, **a**), healthy controls (Healthy, **b**). Extracorporeal membrane oxygenation (ECMO) patients on day 1 (**c**), day 3 (**d**) and after ECMO explantation (“Post”, **e**), Mepacrine stained platelets were stimulated with 0.4 U/ml thrombin and the reduction in platelet MFI was recorded for 20 min after thrombin stimulation using flow cytometry. Instead of thrombin, a control sample was treated with phosphate buffered saline (PBS) as negative control. Results are presented as percentage of baseline MFI. Baseline was defined as the mean fluorescence intensity of mepacrine stained platelets before thrombin or PBS was added. Mepacrine stained platelets from all groups showed a significant reduction in percentage of baseline MFI after 20 min thrombin stimulation compared to PBS treatment indicating that thrombin stimulation led to release of platelet delta granules. Thrombin treatment of mepacrine stained platelets from ECMO patients at different time points showed similar effects (**f**). The number of ECMO patients analyzed at each time point is indicated, as are the number of controls. Data are presented as mean±standard deviation, ***p<0.001

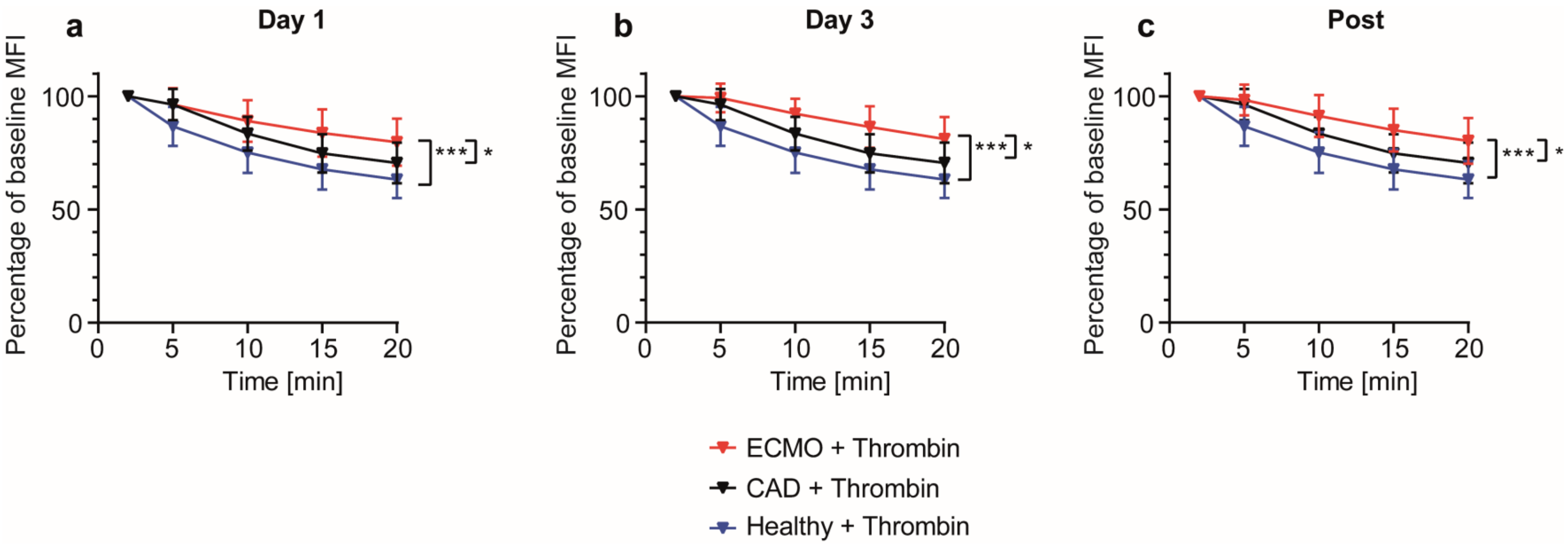

Supplementary Figure S4 Platelet mepacrine release after thrombin stimulation in ECMO patients compared to controls. The fluorescent dye mepacrine is taken up by platelets and stored in delta granules. Thrombin stimulation leads to release of delta granules, which can be quantified by a reduction in platelet mean fluorescence intensity (MFI). The following treatment groups are shown: Extracorporeal membrane oxygenation (ECMO) patients on day 1 (**a**), day 3 (**b**) and after ECMO explantation (‘Post’, **c**), patients with coronary artery disease (CAD) and healthy controls (Healthy). Mepacrine stained platelets were stimulated with 0.4 U/ml thrombin and reduction in platelet mean fluorescence intensity (MFI) was recorded for 20 min after thrombin stimulation using flow cytometry. Results are presented as percentage of baseline MFI. The number of ECMO patients analyzed at each time point is indicated, as is the number of controls. Data are presented as mean±standard deviation. *$p<0.05$, ***$p<0.001$

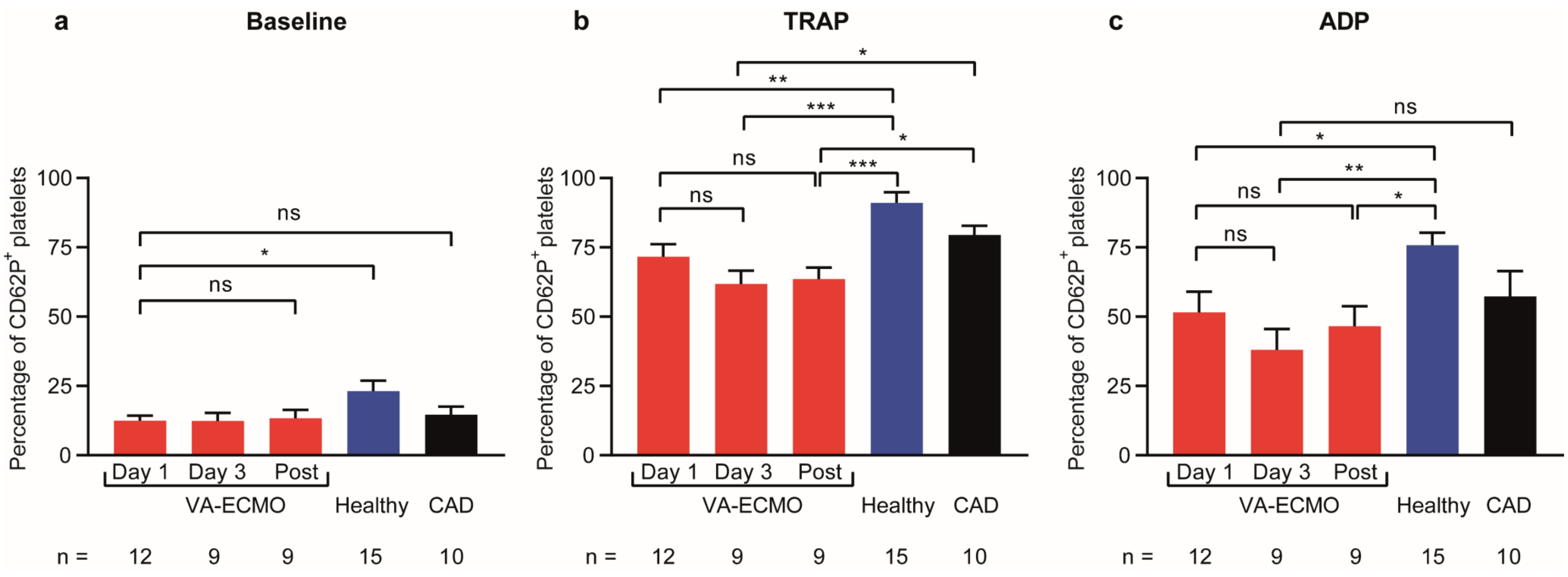

Supplementary Figure S5 Platelet expression of CD62P in patients receiving *veno-arterial extracorporeal membrane oxygenation (VA-ECMO)* compared to controls. Only VA-ECMO patients with known coronary artery disease are presented. CD62P expression was analyzed by flow cytometry on resting (baseline), thrombin receptor activating peptide (TRAP)-stimulated and adenosine diphosphate (ADP)-stimulated platelets. Blood was sampled from VA-ECMO patients on day 1, day 3 and after VA-ECMO explantation (Post). CD62P expression on platelets was compared to healthy controls (Healthy) and patients with coronary artery disease (CAD). **a**, Baseline CD62P expression, CD62P expression in response to TRAP (**b**) and ADP (**c**) stimulation. The number of VA-ECMO patients remaining at each time point and the number of control patients are indicated below. Data are presented as mean±standard error of the mean. *p<0.05, **p<0.01, ***p<0.001

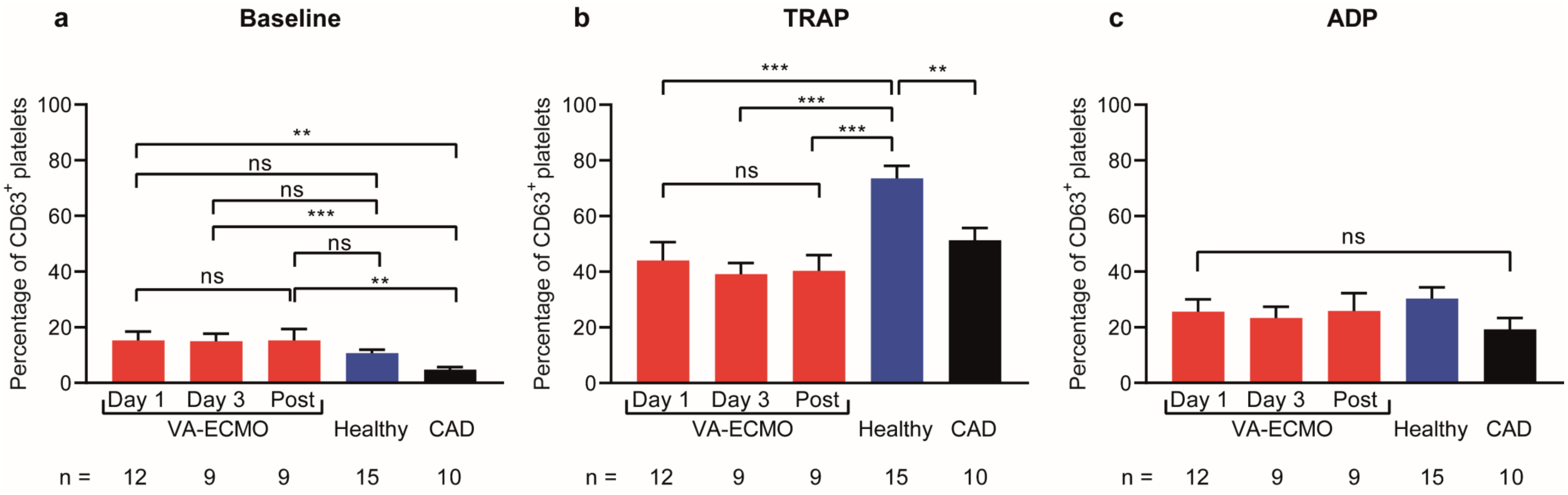

Supplementary Figure S6 Platelet CD63 expression in patients receiving *veno-arterial extracorporeal membrane oxygenation (VA-ECMO)* compared to controls. Only VA-ECMO patients with known coronary artery disease are presented. CD63 expression was analyzed by flow cytometry on resting (baseline), thrombin receptor activating peptide (TRAP)-stimulated and adenosine diphosphate (ADP)-stimulated platelets. Blood was sampled from VA-ECMO patients on day 1, day 3 and after ECMO explantation (Post). CD63 expression on platelets was compared to healthy controls (Healthy) and patients with stable coronary artery disease (CAD). **a**, Baseline CD63 expression was significantly elevated on platelets from VA-ECMO patients compared to CAD. **b**, CD63 expression in response to TRAP was lower in VA-ECMO patients compared to healthy controls. **c**, CD63 expression in response to ADP stimulation was similar in VA-ECMO patients and controls. The number of VA-ECMO patients remaining at each time point and the number of control patients are indicated below. Data are presented as mean±standard error of the mean. ns, not significant, **p<0.01, ***p<0.001

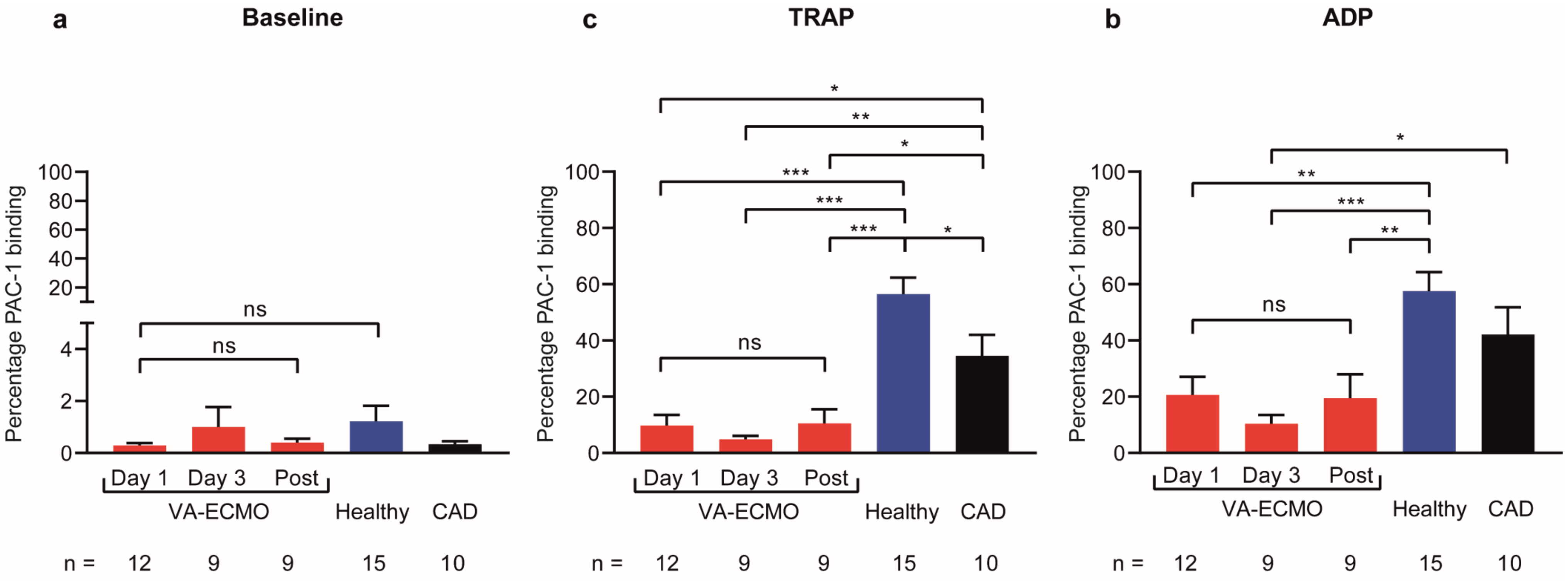

Supplementary Figure S7 Expression of activated GPIIb/IIIa in patients receiving *veno-arterial extracorporeal membrane oxygenation (VA-ECMO)* compared to controls. Only VA-ECMO patients with known coronary artery disease are presented. Activated GPIIb/IIIa expression was analyzed by flow cytometry on resting (baseline), thrombin receptor activating peptide (TRAP)-stimulated and adenosine diphosphate (ADP)-stimulated platelets using the conformation specific antibody PAC-1. Blood was sampled from VA-ECMO patients on day 1, day 3 and after ECMO explantation (Post). Expression of activated GPIIb/IIIa on platelets was compared to healthy controls (Healthy) and patients with coronary artery disease (CAD). **a**, Baseline expression of activated GPIIb/IIIa. **b**, Expression of activated GPIIb/IIIa in response to TRAP stimulation. **c**, Expression of activated GPIIb/IIIa in response to ADP stimulation. The number of VA-ECMO patients remaining at each time point and the number of control patients are indicated below. Data are presented as mean±standard error of the mean. ns, not significant, *p<0.05, **p<0.01, ***p<0.001

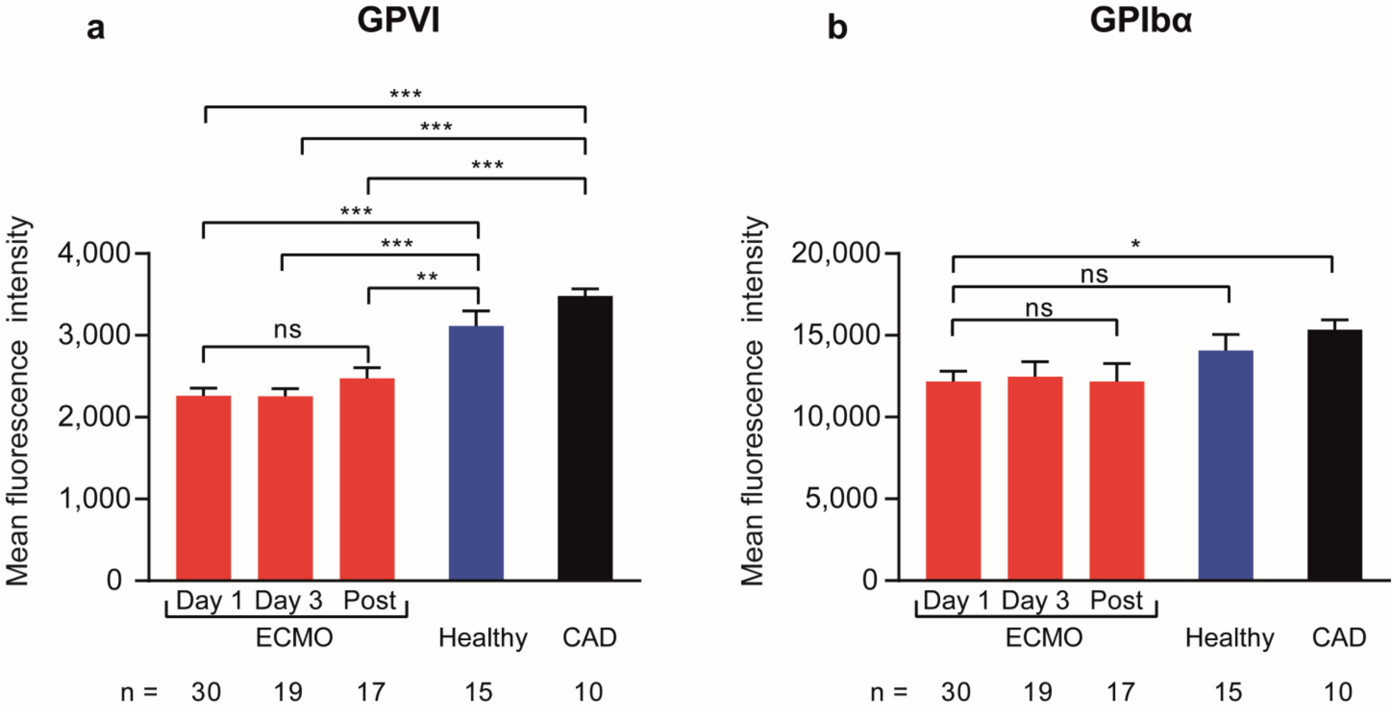

Supplementary Figure S8 Expression of GPVI and GPIbα on resting platelets from ECMO patients compared to controls. Blood was sampled from ECMO patients (ECMO) on day 1, day 3 and after ECMO explantation (Post). Expression of GPVI and GPIbα on platelets was compared to healthy controls (Healthy) and patients with coronary artery disease (CAD). **a**, Expression of GPVI on platelets from ECMO patients was significantly lower at all time points compared to healthy controls and CAD patients. **b**, Expression of GPIbα was also lower compared to controls and significance was achieved on day 1 of ECMO compared to CAD patients. The number of ECMO patients remaining at each time point and the number of control patients are indicated below. Data are presented as mean±SEM. ns, not significant, *p<0.05, **p<0.01, ***p<0.001

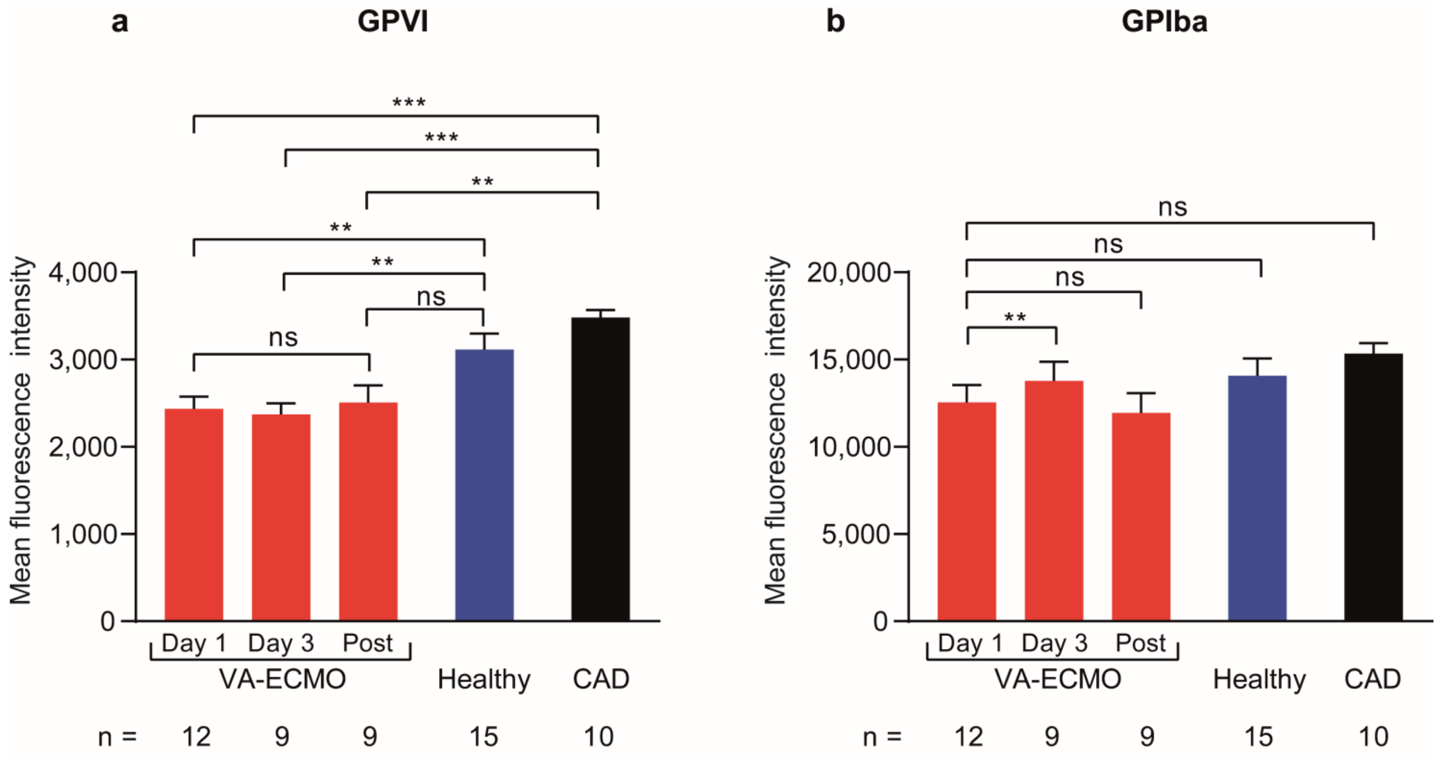

Supplementary Figure S9 Expression of GPVI and GPIbα on resting platelets from patients receiving *veno-arterial extracorporeal membrane oxygenation (VA-ECMO)* compared to controls. Only VA-ECMO patients with known coronary artery disease are presented. Blood was sampled from VA-ECMO patients on day 1, day 3 and after VA-ECMO explantation (Post). Expression of GPVI and GPIbα on platelets was compared to healthy controls (Healthy) and patients with stable coronary artery disease (CAD). **a**, Expression of GPVI; **b**, expression of GPIbα. The number of VA-ECMO patients remaining at each time point and the number of control patients are indicated below. Data are presented as mean±standard error of the mean. ns, not significant, **p<0.01, ***p<0.001

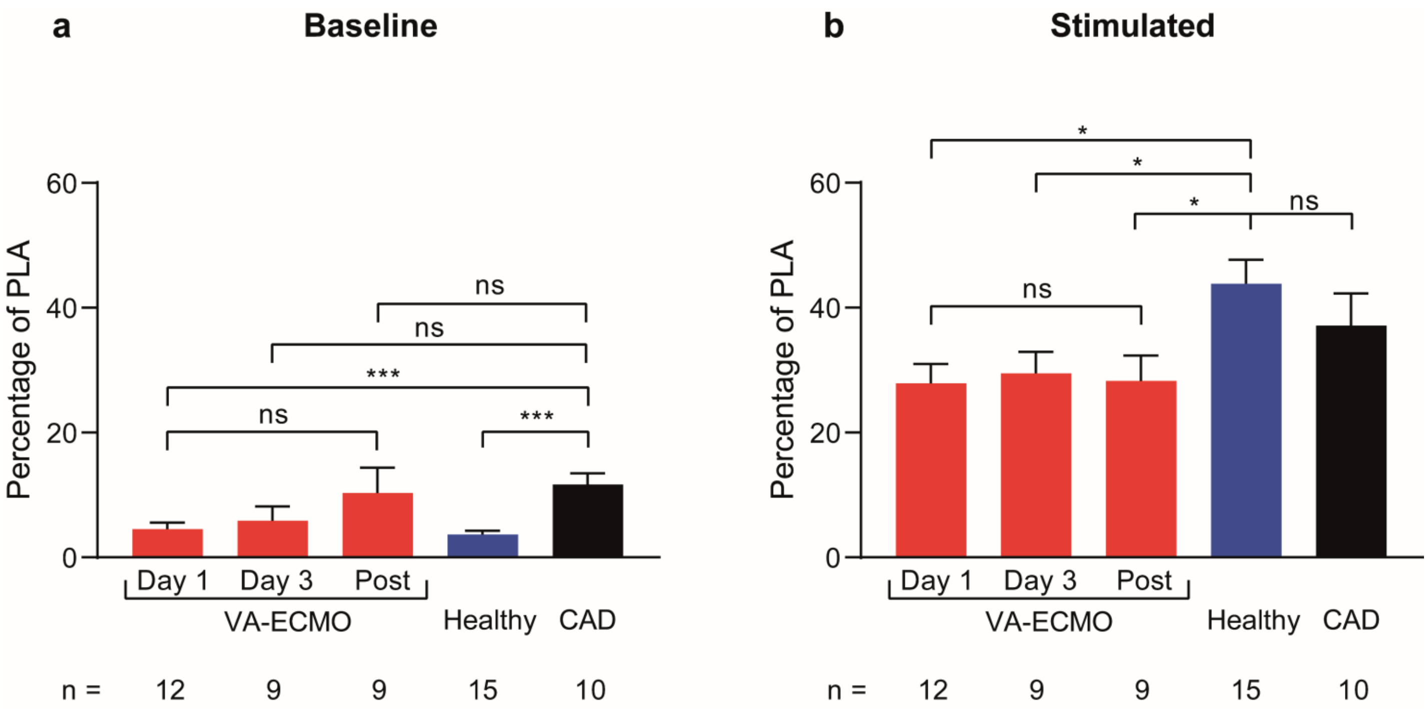

Supplementary Figure S10 Levels of platelet leukocyte aggregates (PLA) in patients *receiving veno-arterial extracorporeal membrane oxygenation (VA-ECMO)* compared to controls. Only VA-ECMO patients with known coronary artery disease are presented. The percentage of CD61+/CD45+ of all CD45+ leukocytes is presented. PLA were analyzed at rest (baseline) and in response to simulation with adenosine diphosphate (ADP) and phorbol 12-myristate 13-acetate (stimulated). Blood was sampled from VA-ECMO patients on day 1, day 3 and after VA-ECMO explantation (Post). PLA in VA-ECMO patients were compared to healthy controls (Healthy) and patients with stable coronary artery disease (CAD). **a**, baseline PLA levels were similar in VA-ECMO patients and healthy controls. CAD patients had higher levels of PLA. **b**, PLA formation in response to stimulation was significantly reduced in VA-ECMO patients and reached significance at all time points compared to healthy controls. The number of VA-ECMO patients remaining at each time point and the number of control patients are indicated below. Data are presented as mean±standard error of the mean. ns, not significant, *p<0.05, ***p<0.001
