# Supplementary material for: Early platelet dysfunction in patients receiving extracorporeal membrane oxygenation is associated with mortality
Source: J Thromb Thrombolysis. 2021 Sep 16;53(3):712–21. doi: 10.1007/s11239-021-02562-9 (PMC8444511; doi:10.1007/s11239-021-02562-9)
Supplement: Supplementary file 3 — Supplementary material 3 (PDF 229.0 kb) [file 11239_2021_2562_MOESM3_ESM.pdf]

Early platelet dysfunction in patients receiving extracorporeal membrane oxygenation is associated with mortality

Journal of Thrombosis and Thrombolysis

Patrick M. Siegel, MD[1]; Julia Chalupsky, M.Sc.[1]; Christoph B. Olivier, MD[1]; István Bojti, MD[1]; Jan-Steffen Pooth, MD[2]; Georg Trummer, MD[2]; Christoph Bode, MD[1]; Philipp Diehl, MD, PhD[1]

[1] Department of Cardiology and Angiology I, University Heart Center Freiburg – Bad Krozingen, Faculty of Medicine, University of Freiburg, Freiburg, Germany

[2] Department of Cardiovascular Surgery, University Heart Center Freiburg – Bad Krozingen, Faculty of Medicine, University of Freiburg, Freiburg i. Br., Germany

Corresponding author

Dr. Patrick Malcolm Siegel

Department of Cardiology and Angiology I, Heart Center Freiburg University

Faculty of Medicine, University of Freiburg

Freiburg, 79106

Germany

Tel: +49 761/270-34010

E-Mail: patrick.siegel@universitaets-herzzentrum.de

Parameter	VA-ECMO patients
Patients, n (%)	12 (100)
Age, y (Q1-Q3)	69 (60-74)
Female, n (%)	2 (17)
Survivors, n (%)	8 (67)
ECMO system, n (%)	
Stöckert Sorin	6 (50)
Maquet	4 (33)
Deltastream	1 (8)
CARL	1 (8)
Days on ECMO (median, Q1-Q3)	6.5 (4.0-7.0)
ECMO blood flow on day 1 (l/min, Q1-Q3)	4.5 (3.8-4.9)
Indications for VA-ECMO, n (%)	
Cardiogenic shock	9 (75)
eCPR	3 (25)
Coronary artery disease, n (%)	12 (100)
Severe valvular heart disease, n (%)	1 (8)
Atrial fibrillation, n (%)	1 (8)
Diabetes mellitus, n (%)	1 (8)
Hypertension, n (%)	0 (0)
Smoking, n (%)	2 (17)
Hypercholesterolemia, n (%)	3 (25)
Acute renal failure during ECMO, n (%)	3 (25)
Acute liver failure during ECMO, n (%)	1 (8)
Major bleeding during ECMO, n (%)	6 (50)
Thrombotic events during ECMO, n (%)	1 (8)
Received Heparin during ECMO, n (%)	12 (100)
Received ASA during ECMO, n (%)	11 (92)
Received ASA + P_2Y_{12} inhibitor during ECMO, n (%)	7 (58)
Received transfusions during ECMO, n (%)	9 (75)
Mechanical ventilation on day 1, n (%)	12 (100)
SOFA score on day 1 (Q1-Q3)	11.0 (10.0-14.0)
WBC (x10^3/µl, Q1-Q3)	9.0 (5.3-10.7)
Platelets (x10^3/µl, Q1-Q3)	120.5 (91.0-183.0)
Hemoglobin (g/dl, Q1-Q3)	8.7 (8.2-10.7)
Creatinine (mg/dl, Q1-Q3)	1.7 (1.0-2.5)

Urea (mg/dl, Q1-Q3)	54.0 (36.0-60.3)
Bilirubin (mg/dl, Q1-Q3)	1.9 (1.1-2.8)
AST (U/l, Q1-Q3)	249.0 (98.3-622.5)
ALT (U/l, Q1-Q3)	69.0 (31.3-234.0)
CRP (mg/l, Q1-Q3)	29.8 (15.8-99.9)
LDH (U/l)	640.5 (380.5-1306)
PTT (s, Q1-Q3)	46.0 (37.3-58.3)
INR (Q1-Q3)	1.3 (1.0-1.4)
Fibrinogen (mg/dl)	203.0 (158.0-295.0)
Lactate (mmol/l, Q1-Q3)	3.4 (2.4-5.2)
p_aO_2 (mmHg, Q1-Q3)	126.5 (95.2-254.5)
p_aCO_2 (mmHg, Q1-Q3)	40.7 (36.0-41.6)
F_iO (%, Q1-Q3)	50.0 (40.0-50.0)
PEEP (mbar, Q1-Q3)	9.0 (7.0-14.0)
Respiratory rate (/min, Q1-Q3)	14.0 (11.3-16.0)

Supplementary Table S1 Clinical characteristics of VA-ECMO patients with coronary artery disease. The results are presented as median (interquartile range, Q1-Q3) for continuous variables, and number (percentage) for categorical variables. Denominator of the percentage is the total number of subjects in the group. Laboratory parameters and ventilation settings presented were taken from the patient data management system on day 1 closest to the time of blood sampling for platelet function analysis. ALT, alanine aminotransferase; ARDS, acute respiratory distress syndrome; AST, aspartate aminotransferase; CRP, C-reactive protein; eCPR, extracorporeal cardiopulmonary resuscitation; F_iO, fraction of inspired oxygen; INR, international normalized ratio; LDH, lactate dehydrogenase; VA-ECMO, veno-arterial extracorporeal membrane oxygenation; VV-ECMO, veno-venous extracorporeal membrane oxygenation. p_aO_2, partial oxygen pressure in arterial blood; p_aCO_2, partial pressure of carbon dioxide in arterial blood; PEEP, positive endexpiratory pressure; PTT, partial thromboplastin time; SOFA, sequential organ failure assessment; WBC, white blood cells.

Parameter	CAD
Patients, n (%)	10 (100)
Age, y (Q1-Q3)	67.0 (51.5-76.5)
Female, n (%)	4 (40)
Coronary artery disease, n (%)	10 (100)
Atrial fibrillation, n (%)	5 (50)
Diabetes mellitus, n (%)	5 (50)
Hypertension, n (%)	8 (80)
Smoking, n (%)	4 (40)
Hypercholesterolemia, n (%)	9 (90)
LVEF, % (Q1-Q3)	55 (50-55)
Received ASA, n (%)	7 (70)
Received ASA + P_2Y_{12} inhibitor (%)	6 (60)
Vitamin K antagonists, n (%)	1 (10)
Direct Factor Xa antagonists, n (%)	4 (40)
WBC ($x10^3$ /µl, Q1-Q3)	7.7 (6.2–8.5)
Platelets ($x10^3$ /µl, Q1-Q3)	235 (209.5-269.8)
Hemoglobin (g/dl, Q1-Q3)	13.9 (12.6-15.4)
Creatinine (mg/dl, Q1-Q3)	1.0 (0.8-1.1)
Urea (mg/dl, Q1-Q3)	33.0 (29.8-41.3)
AST (U/l, Q1-Q3)	24.5 (21.3-30.0)
ALT (U/l, Q1-Q3)	21.0 (16.5-64.0)
CRP (mg/l, Q1-Q3)	8.4 (3.8-12.6)
LDH (U/l)	216 (182.0-291.3)
aPTT (s, Q1-Q3)	29.0 (27.5-35.5)
INR	1.1 (1.0-1.3)

Supplementary Table S2. Clinical characteristics of control patients with coronary artery disease (CAD). Data are presented as median (interquartile range, i.e. Q1-Q3) for continuous variables, and number (percentage) for categorical variables. Denominator of the percentage is the total number of subjects in the group. Laboratory values on the day of coronary angiography are presented and were taken from the clinical data management system. Medication is stated as received on the day of coronary angiography except for direct factor Xa antagonists, which were not given to patients as per hospital protocol on the day of coronary angiography to reduce bleeding risk. ALT, alanine aminotransferase; ASA, acetylsalicylic acid; AST, aspartate aminotransferase; CRP, C-reactive protein; F_iO, fraction of inspired oxygen;

INR, international normalized ratio; LDH, lactate dehydrogenase; LVEF, left ventricular ejection fraction; p_aO_2, partial oxygen pressure in arterial blood; $p_aCO_{2,}$ partial pressure of carbon dioxide in arterial blood; PTT, partial thromboplastin time; PEEP, positive endexpiratory pressure; WBC, white blood cells.

Parameter	Survivors	Non-Survivors
Patients, n (%)	15 (100)	15 (100)
Age, y (Q1-Q3)	64.0 (58.0-71.0)	59.0 (44.0-71.0)
Female, n (%)	4 (27)	3 (20)
VV-ECMO, n (%)	3 (20)	7 (47)
VA-ECMO, n (%)	12 (80)	8 (53)
ECMO system, n (%)		
Stöckert Sorin	6 (40)	6 (40)
Maquet	5 (33)	8 (53)
Deltastream	3 (20)	1 (7)
CARL	1 (7)	0 (0)
Days on ECMO (median, Q1-Q3)	7.0 (4.0-7.0)	4.0 (3.0-8.0)
ECMO blood flow on day 1 (l/min, Q1-Q3)	4.4 (3.4-4.9)	4.0 (3.1-5.0)
Indications for VA-ECMO, n (%)		
Cardiogenic shock	11 (73)	6 (40)
- Postoperative	7 (47)	2 (13)
- Myocardial Infarction	1 (7)	1 (7)
- Cardiomyopathy	0 (0)	1 (7)
- Post cardiac arrest	2 (13)	1 (7)
- Endocarditis	0 (0)	1 (7)
- Pulmonary Embolism	1 (7)	0 (0)
eCPR	1 (7)	2 (13)
Indications for VV-ECMO, n (%)		
ARDS	3 (20)	6 (40)
- Primary	2 (13)	5 (33)
- Secondary	1 (7)	1 (7)
Pulmonary Embolism	0 (0)	1 (7)
Coronary artery disease, n (%)	8 (53)	4 (27)
Severe valvular heart disease, n (%)	3 (20)	2 (13)

Atrial fibrillation, n (%)	4 (27)	3 (20)
Diabetes mellitus, n (%)	1 (7)	2 (13)
Hypertension, n (%)	1 (7)	1 (7)
Smoking, n (%)	2 (13)	1 (7)
Hypercholesterolemia, n (%)	3 (20)	1 (7)
Cancer, n (%)	0 (0)	1 (7)
Acute renal failure during ECMO, n (%)	3 (20)	10 (67)
Acute liver failure during ECMO, n (%)	0 (0)	3 (20)
Major bleeding during ECMO, n (%)	4 (27)	11 (73)
Thrombotic events during ECMO, n (%)	3 (20)	0 (0)
Received Heparin during ECMO, n (%)	14 (93)	12 (80)
Received ASA during ECMO, n (%)	10 (67)	5 (33)
Received ASA + P_2Y_{12} inhibitor during ECMO, n (%)	4 (27)	3 (20)
Received transfusions during ECMO, n (%)	8 (53)	10 (67)
Mechanical ventilation on day 1, n (%)	15 (100)	15 (100)
SOFA score on day 1 (Q1-Q3)	10.0 (9.0-12.0)	13.0 (11.0-16.0)
WBC (x10^3 /µl, Q1-Q3)	8.8 (6.0-10.5)	17.0 (6.6-21.1)
Platelets (x10^3 /µl, Q1-Q3)	126.0 (97.0-184.0)	61.0 (43.0-200.0)
Hemoglobin (g/dl, Q1-Q3)	8.9 (8.4-11.0)	8.7 (8.1-10.0)
Creatinine (mg/dl, Q1-Q3)	1.0 (0.9-2.2)	2.0 (1.0-2.6)
Urea (mg/dl, Q1-Q3)	42.0 (32.3-65.8)	58.0 (40.0-96.0)
Bilirubin (mg/dl, Q1-Q3)	1.6 (1.1-2.7)	3.4 (1.3-4.2)
AST (U/l, Q1-Q3)	117 (52.5-233.5)	257 (98.0-1700.0)
ALT (U/l, Q1-Q3)	41.5 (28.0-93.8)	76.0 (52.3-463.0)
CRP (mg/l, Q1-Q3)	54.1 (17.5-159.3)	108.1 (36.0-260.6)
LDH (U/l)	406.0 (390.0-643.0)	957.0 (400.3-2616)
PTT (s, Q1-Q3)	43.0 (38.0-52.8)	51.5 (38.5-96.0)
INR	1.2 (1.1-1.3)	1.3 (1.1-1.6)
Fibrinogen (mg/dl)	262.5 (165.0-491.8)	327.0 (147.8-557.0)

Lactate (mmol/l, Q1-Q3)	2.8 (1.2-3.7)	2.6 (1.4-5.5)
p_aO_2 (mmHg, Q1-Q3)	107.0 (80.2-132.0)	104.0 (63.40-205.0)
p_aCO_2 (mmHg, Q1-Q3)	41.4 (39.2-44.0)	37.2 (33.2-42.4)
F_iO (%, Q1-Q3)	40.0 (40-50)	50.0 (45.0-60.0)
PEEP (mbar, Q1-Q3)	8.0 (7.0-10.0)	15.0 (11.0-19.0)
Respiratory rate (/min, Q1-Q3)	15.0 (12.0-16.0)	17.0 (12.0-21.0)

Supplementary Table S3. Clinical characteristics of survivors and non-survivors. The results are presented as median (interquartile range, Q1-Q3) for continuous variables, and number (percentage) for categorical variables. Denominator of the percentage is the total number of subjects in the group. ARDS, acute respiratory distress syndrome; ALT, alanine aminotransferase; ASA, acetylsalicylic acid; AST, aspartate aminotransferase; CRP, C-reactive protein; eCPR, extracorporeal cardiopulmonary resuscitation; FiO, fraction of inspired oxygen; p_aO_2, partial oxygen pressure in arterial blood; p_aCO_2, partial pressure of carbon dioxide in arterial blood; PTT, partial thromboplastin time; PEEP, positive endexpiratory pressure; SOFA – sequential organ failure assessment; VA-ECMO, veno-arterial extracorporeal membrane oxygenation; VV-ECMO, veno-venous extracorporeal membrane oxygenation; WBC, white blood cells.
